# A shelf stable Fmoc hydrazine resin for the synthesis of peptide hydrazides

**DOI:** 10.1002/pep2.24268

**Published:** 2022-04-16

**Authors:** Michael J. Bird, Philip E. Dawson

**Affiliations:** ^1^ Department of Chemistry The Scripps Research Institute La Jolla California USA

**Keywords:** Fmoc resins, native chemical ligation, peptide hydrazides, peptide thioesters, solid phase peptide synthesis

## Abstract

C‐terminal hydrazides are an important class of synthetic peptides with an ever expanding scope of applications, but their widespread application for chemical protein synthesis has been hampered due to the lack of stable resin linkers for synthesis of longer and more challenging peptide hydrazide fragments. We present a practical method for the regeneration, loading, and storage of trityl‐chloride resins for the production of hydrazide containing peptides, leveraging 9‐fluorenylmethyl carbazate. We show that these resins are extremely stable under several common resin storage conditions. The application of these resins to solid phase peptide synthesis (SPPS) is demonstrated through the synthesis of the 40‐mer GLP‐1R agonist peptide “P5”. These studies support the broad utility of Fmoc‐NHNH‐Trt resins for SPPS of C‐terminal hydrazide peptides.

## Graphical abstract

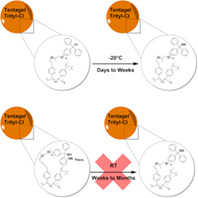

## 1 INTRODUCTION

The hydrazide moiety has been ubiquitous in the field of peptide chemistry for more than 50 years,^[^ ^1^, ^2^, ^3^, ^4^, ^5^, ^6^, ^7^, ^8^, ^9^, ^10^, ^11^, ^12^, ^13^, ^14^, ^15^, ^16^, ^17^ ^]^ primarily due to its utility as a precursor to acyl azides, used for amide couplings^[^ ^1^, ^2^, ^3^, ^4^, ^5^, ^6^, ^7^, ^8^, ^9^, ^10^, ^11^, ^12^, ^13^, ^18^ ^]^ and thioester formation^[^ ^14^, ^15^, ^16^, ^17^ ^]^ to enable Native Chemical Ligation.^[^ ^19^ ^]^ Our group recently published mild conditions for formation of peptide α‐thioesters from hydrazides via the Knorr pyrazole synthesis.^[^ ^17^ ^]^ This method avoids the need for strong oxidizing conditions, enabling broader compatibility with common synthetic peptide functionalities, and has been widely adopted in a broad range of applications.^[^ ^20^, ^21^, ^22^, ^23^, ^24^, ^25^, ^26^, ^27^, ^28^, ^29^, ^30^, ^31^, ^32^, ^33^, ^34^, ^35^, ^36^, ^37^ ^]^

The increased use of C‐terminal α‐hydrazides in peptide chemistry creates a demand for robust synthetic tools for accessing these moieties. A number of methods have been published to allow access to C‐terminal hydrazides on both synthetic^[^ ^15^, ^16^, ^38^ ^]^ and expressed^[^ ^15^, ^39^, ^40^ ^]^ peptides and proteins. The current synthetic methods suffer from one of two drawbacks. They either require a two part deprotection procedure involving hydrazinolysis followed by global deprotection,^[^ ^38^ ^]^ or rely on time‐of‐use formation of hydrazine resins from trityl chloride resins, where the hydrazine form is not shelf stable.^[^ ^16^ ^]^ The chloride form may also need to be regenerated prior to use, making solid phase peptide synthesis (SPPS) of peptide hydrazides significantly less convenient than standard amides or acids.^[^ ^41^, ^42^, ^43^ ^]^

**FIGURE 1:**
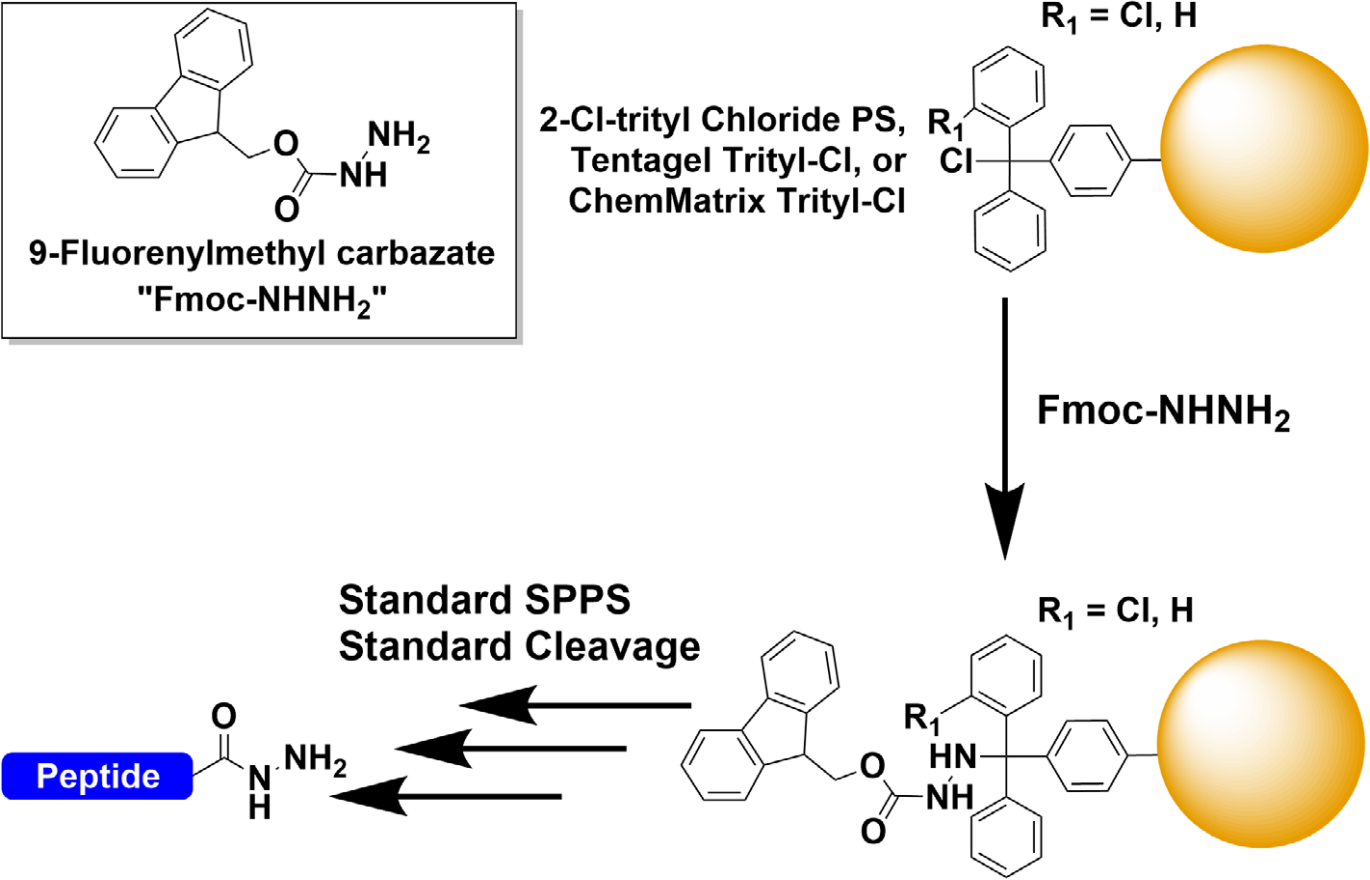
Proposed new strategy for preparation of peptide hydrazides using a stable Fmoc‐NHNH_2_ loaded resin precursor

Chemical protein synthesis requires the solid phase synthesis of long and hydrophobic peptide fragments, which has been addressed through the use of higher swelling polyethylene glycol (PEG) containing resins.^[^ ^44^, ^45^, ^46^, ^47^, ^48^ ^]^ When using PEGylated trityl chloride resins (e.g., TentaGel® and ChemMatrix®), the yields of synthetic peptides are often significantly lower than expected and decrease over time after resins are first opened.^[^ ^49^ ^]^ In addition, while there has been a move toward increased automation and real‐time monitoring of peptide synthesis and purification workflows, regenerating and hydrazine loading a trityl chloride resin prior to each synthesis represents a manual process that must be performed in a fume hood, and cannot be monitored other than by initiation of synthesis. We therefore sought to develop a procedure for regenerating and loading trityl chloride resins that, upon receipt from the manufacturer, would allow for immediate loading evaluation and long term stable storage in a state that is competent for automated synthesis of peptide α‐hydrazides without any extraneous steps. 9‐Fluorenylmethyl carbazate (Fmoc‐NHNH_2_) was identified as an ideal reagent for preparation and long term storage of peptide‐hydrazide synthesis resins (Figure 1), and the relative stability and regeneratability of TentaGel® resins loaded with Fmoc‐NHNH_2_ as compared to chloride or hydrazine was explored.

## 2 MATERIALS AND METHODS

### 2.1 Materials

N,N‐dimethylformamide (DMF), dichloromethane (DCM), acetonitrile (ACN), Oxyma Pure, ethanedithiol (EDT), 2‐methyltetrahydrofuran (2‐MeTHF), and trifluoroacetic acid (TFA) were purchased from Sigma‐Aldrich. Diisopropylethylamine (DIEA), 1‐[bis(dimethylamino)methylene]‐1H‐1,2,3‐triazolo[4,5‐b]pyridinium‐3‐oxidhexafluorophosphate (HATU), diisopropylcarbodiimide (DIC), Fmoc‐NHNH_2_, and triisopropylsilane (TIS) were purchased from Oakwood Chemical. 2′‐Chlorotritylchloride polystyrene resin (200–400 mesh) and all standard Fmoc‐protected amino acids were purchased from Bachem Americas. 4‐Methylpiperidine (4‐MePip) was purchased from TCI America. Dimethyl sulfide (DMS) and 4‐dimethylaminopyridine (DMAP) were purchased from AlfaAesar. TentaGel® XV trityl chloride resin (XV18130.031) was purchased from Rapp Polymere. Trityl‐OH ChemMatrix® (7–600‐1310) was purchased from Biotage. Diethyl ether (Et_2_O) and septa capped vials for storage under inert gas were purchased from Fisher Scientific. Methyl tert‐butyl ether (MTBE) was purchased from VWR.

### 2.2 Chloride regeneration and Fmoc‐NHNH_2_ loading

TentaGel® XV Trityl Chloride was swollen in minimal DCM within a round bottom flask fitted with a rubber septum or a 40 ml glass vial fitted with a 0.125″ septa cap. The headspace was purged and backfilled with nitrogen. Five equiv of thionyl chloride, calculated from manufacturer reported resin loading, was added and the reaction was stirred under nitrogen for 2.5 h. The slurry was transferred to a fritted syringe and washed with DCM then DMF. The resin was treated with 5 equiv of Fmoc‐NHNH_2_ suspended in 50:50 DCM:DMF to barely cover the swelled resin and stirred for 45 min. The resin was drained and rinsed with DMF, then the Fmoc‐NHNH_2_ treatment was repeated. The resin was flow washed with DMF and DCM and dried by flow washing with Et_2_O over a vacuum manifold before storing in a vacuum desiccator for at least 8 h prior to performing loading tests. Thionyl chloride, SOCl_2_, is requires careful handling for safety. SOCl_2_ causes severe burns on skin contact and should only be used in a fume hood due to inhalation toxicity. Appropriate PPE including gloves, lab coat, and eye protection should be worn at all times. Care should be taken to avoid mixing of thionyl chloride with water, with which it reacts violently. SOCl_2_ must be disposed of in appropriate hazardous waste containers, as it represents a potential environmental hazard. If needles are used during transfer of DCM or thionyl chloride, extreme care should be taken to avoid accidental skin pricks, which can cause severe damage, and require immediate medical attention. Washing with Et_2_O is integral to proper resin drying (see Supporting information), but care should be taken to regularly dispose of any ether containing waste to avoid the buildup of potentially explosive peroxides. Bottles of Et_2_O should be opened and used promptly, and hazardous waste specialists should be consulted before disposal or handling of any expired containers, or containers where a buildup of brown solid is observed.

### 2.3 Fmoc loading tests

Fmoc loading tests were performed in triplicate by taking single absorbance measurement on three independently weighed and treated samples. Approximately 4 μmol of resin was weighed into a 1.7 ml microcentrifuge tube. For example, for a TentaGel® XV resin with an estimated loading of 0.2 mmol/g, 20 mg of resin were used. Each sample was suspended in 1 ml of 20% 4‐MePip in DMF and sonicated to ensure full resin swelling, then mixed on a rotating shaker for 20 min. The tubes were centrifuged and 10 μl of supernatant was diluted to 1 ml with DMF. The spectrometer was blanked with 0.2% 4‐MePip in DMF and *A*_301_ measurements were taken for each sample. Loadings were calculated according to the formula $\frac{101\left(A_{301}\right)}{7.8\left(w\right)}$, where *w* is the weight of the resin used in milligrams.^[^ ^50^ ^]^ Reported loadings are the arithmetic mean of the triplicate loading tests ± 1 standard deviation, and error bars in figures represent ± 1 standard deviation.

### 2.4 Peptide synthesis and cleavage

All peptides were synthesized by conventional Fmoc/tBu chemistry using either [5 equiv amino acid]:[5 equiv HATU]:[8 equiv DIEA] or [5 equiv amino acid]:[5 equiv DIC]:[5 equiv Oxyma Pure] on a CSBio model CS336X Peptide Synthesizer. Peptides were cleaved from the resin in one of four cocktails according to the peptide composition:

Cocktail N: Peptides containing neither cysteine nor methionine

- 95% TFA
- 2.5% TIS
- 2.5% H_2_O

Cocktail C: Peptides containing cysteine but not methionine

- 92.5% TFA
- 2.5% TIS
- 2.5% H_2_O
- 2.5% EDT

Cocktail M: Peptides containing methionine but not cysteine

- 87.5% TFA
- 5.0% TIS
- 2.5% H_2_O
- 5.0% DMS

Cocktail MC: Peptides containing methionine and cysteine

- 85% TFA
- 5.0% TIS
- 2.5% H_2_O
- 2.5% EDT
- 5.0% DMS.

Cleavage reactions were carried out for 1 h at room temperature and TFA removed by nitrogen flow or rotary evaporation. Crude peptide was recovered by precipitation in diethyl ether. TFA is a corrosive and hazardous acidic reagent that fumes profusely under standard atmospheric conditions. It should only be used in a properly ventilated fume hood alongside proper gloves, lab coats, and eye protection. It represents an environmental hazard and must not be disposed of in drains. While evaporation under a nitrogen stream has been a common technique, the use of rotary evaporation followed by appropriate hazardous waste disposal reduces the environmental impact of TFA usage.

### 2.5 Peptide characterization and purification

Peptides were characterized on a Waters Acquity I‐Class UPLC equipped with a diode array detector and a single‐quadrupole mass spectrometer (Waters SQD2). The analysis was performed on a Waters Cortecs C18 column (2.1 × 55 mm, 1.6 μm, 90 Å) heated to 35°C with a 0.8 ml/min flow rate (see gradient info below). Peptides masses were manually deconvoluted using the experimental mass to charge ratios (m/z) from all the observed peptide protonation states by using the onboard analyst software packages.

Alternatively, proteins and longer peptides were characterized on a Waters Acquity I‐Class UPLC equipped with a diode array detector and a time of flight (ToF) mass spectrometer (Waters G2‐XS). The analysis was performed on a Waters BEH C18 column (2.1 × 55 mm, 1.7 μm, 300 Å) heated to 55°C with a 0.4 ml/min flow rate (see gradient info below). Peptides masses were deconvoluted (MaxEnt1 algorithm in Waters MassLynx software) to a monoisotopic singly‐charged mass. In both cases the mobile phases were:

- Buffer A: H_2_O (0.1% Formic Acid)
- Buffer B: MeCN (0.06% Formic Acid)

Peptide purification was carried out by mass directed reversed phase HPLC (RP‐HPLC) on a Waters 2545 Binary Gradient Module equipped with an Acquity QDa Detector and a Waters 2767 Sample Manager. The column was a Waters XBridge Prep C18 and the mobile phases were as above, with the exception that buffer B did not contain Formic Acid.

### 2.6 Storage temperature

The storage temperatures reported are based on readings taken from thermometers in resin storage freezers and refrigerators. Resins stored together in the same freezer were stored in the same part of the freezer to ensure similar conditions. Room temperature varied between 18 and 22°C.

## 3 RESULTS AND DISCUSSION

**FIGURE 2:**
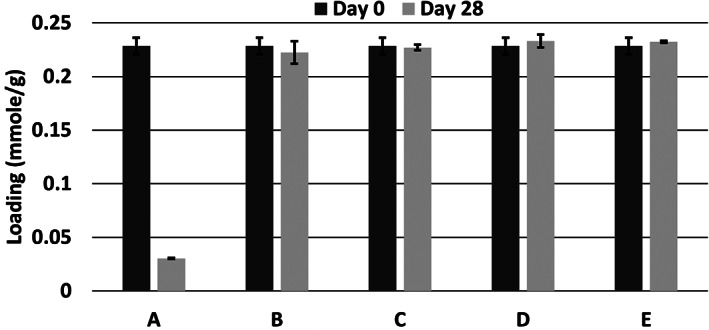
Retention of loading of Tentagel® XV resins stored as: (a) Cl‐Trityl at −10°C sealed under N_2_, (b) Fmoc‐NHNH‐Trityl at −10°C sealed under N_2_, (c) Fmoc‐NHNH‐Trityl at −10°C sealed under air, (d) Fmoc‐NHNH‐Trityl at 3°C sealed under air, and (e) Fmoc‐NHNH‐Trityl at RT open in a vacuum desiccator. Dark gray is day 0, light gray is day 28

Encouraged by preliminary experiments on both Tentagel® XV Trityl and ChemMatrix® Trityl resins (Figure S1, Tables S1 and S2), as well as on 2‐chlorotrityl chloride polystyrene (Figure S2, Table S8), a systematic analysis of loading retention of Fmoc‐NHNH_2_ on the Tentagel® XV was undertaken. To evaluate the stability of carbazate loaded trityl resins, Tentagel® XV trityl chloride with a manufacturer reported loading of 0.19 mmol/g was regenerated with thionyl chloride immediately following receipt from the manufacturer. A sample was taken for storage as the chloride, then the remainder was substituted with Fmoc‐NHNH_2_, and both fractions were dried thoroughly with Et_2_O. The loading of this resin was measured to be 0.228 ± 0.008 mmol/g in a triplicate Fmoc loading test. The dry Fmoc‐NHNH‐Trityl resin was split into four equal fractions and stored in varying conditions for 28 days, alongside the chloride sample which was stored at −10°C under nitrogen atmosphere. The retention of loading was measured by first substituting the chloride resin with Fmoc‐NHNH_2_, then washing all five samples with DMF, DCM, and Et_2_O to remove any Fmoc species not bound to the resin. The resins were then dried from Et_2_O under vacuum and stored in a vacuum desiccator for 8 h. prior to performing Fmoc‐loading tests. Under all conditions tested the Fmoc‐NHNH‐Trityl resins had loadings that were indistinguishable from the day zero, while the chloride resin retained only 13.3% ± 0.3% of its initial loading (Figure 2, Table S3).

In order to establish whether this stability enhancement is unique to the Fmoc‐NHNH_2_ loaded resins as compared to previously reported hydrazide loaded resins^[^ ^16^ ^]^ sample D from Figure 2 (stored at 3°C under air for 28 days) was split in two equal portions and swelled in 1:1 DCM:DMF. One sample (D2) was then drained and deprotected with two, 15 min treatments with 20% 4‐methylpiperidine in DMF, while the other (D1) was left swollen in DCM:DMF. Both were washed thoroughly with DMF, DCM, and Et_2_O before thoroughly drying and storing in a vacuum desiccator for a period of 7 days. Both resins were then swollen, D1 was deprotected, and Fmoc‐glycine was coupled to each resin before thorough washing and drying. The Fmoc‐protected resin (D1) retained a loading of 0.188 ± 0.009 mmol/g, 81% of its initial value, with the losses believed to be caused by incomplete coupling or partial deprotection of the Fmoc‐glycine. Meanwhile, the resin stored as the free hydrazine (D2) had a final loading of 0.0224 ± 0.001 mmol/g, just 9.6% of its initial value (Figure S3, Table S4). This indicates the Fmoc group is required to maintain stable loading of the resin.

**FIGURE 3:**
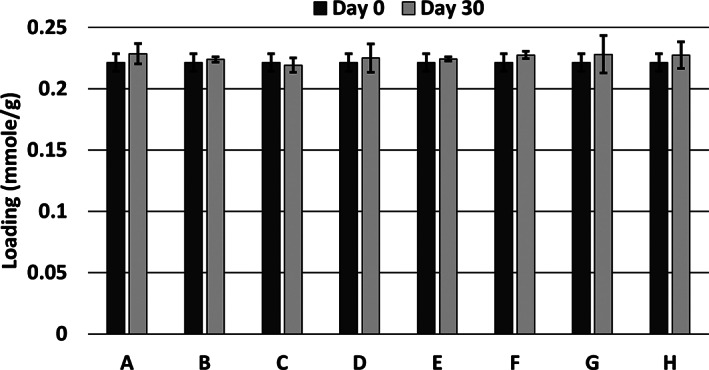
Retention of loading of Tentagel® XV Fmoc‐NHNH‐Trityl resins stored in a −10°C freezer: (a) in DCM, (b) in MTBE, (c) in 2‐MeTHF, (d) in DMF, (e) in 1:1 DCM:DMF, (f) in 2:1 DCM:DMF, (g) dry in air, and (h) dry under N_2_. Dark gray is day 0, light gray is day 30. DCM, dichloromethane; DMF, N,N‐dimethylformamide; 2‐MeTHF, 2‐methyltetrahydrofuran; MTBE, methyl tert‐butyl ether

Another strategy that could help to protect resins from hydrolysis, and that would allow rapid initiation of syntheses would be to maintain pre‐measured and swollen resins. To determine if this is a viable strategy for Fmoc‐NHNH‐Trityl resins a supply of Tentagel® XV trityl chloride with a manufacturer reported loading of 0.22 mmol/g was regenerated and substituted with Fmoc‐NHNH_2_. After thoroughly washing with DMF and DCM and drying with Et_2_O and a vacuum desiccator the loading was measured to be 0.221 ± 0.007 mmol/g. The dry resin was split into eight fractions, all of which were stored together in a −10°C freezer for 30 days. Prior to measuring loading retention each resin was washed with DMF and DCM and dried thoroughly with Et_2_O and 8 h of vacuum desiccation. Triplicate loading tests on each sample showed no loss of loading for any condition (Figure 3, Table S5).

**FIGURE 4:**
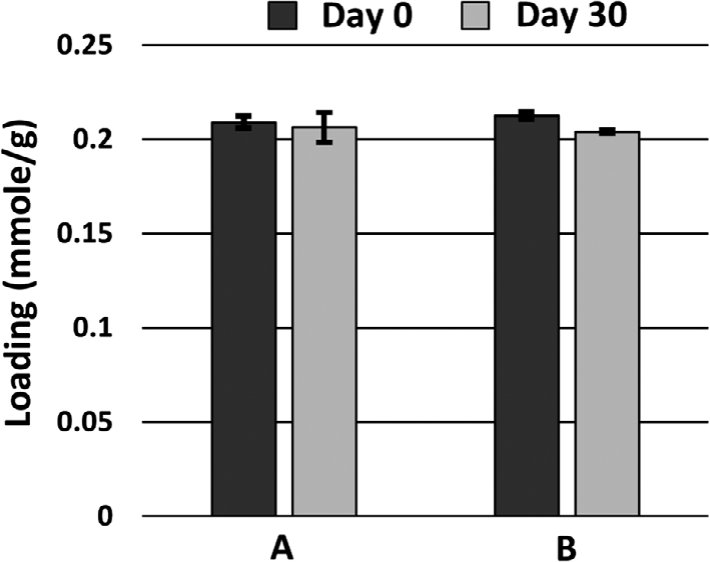
Retention of loading of Tentagel® XV Fmoc‐NHNH‐Trityl resins stored at room temperature, open to air. (a) and (b) Represent two independent resin samples. Dark gray is day 0, light gray is day 30 (7 days in desiccator + 23 days open to air)

We further investigated the stability of the resins at room temperature without vacuum storage. Two separate samples of resin measured at 0.209 ± 0.003 and 0.213 ± 0.002 mmol/g, respectively were stored in a vacuum desiccator for 7 days, then removed and stored, open to air, at room temperature, for a further 23 days. These resins were then thoroughly washed and dried with Et_2_O followed by overnight desiccation, and their loadings were re‐tested in triplicate, showing loading retentions of greater than 95% (Figure 4, Tables S6 and S7).

**FIGURE 5:**
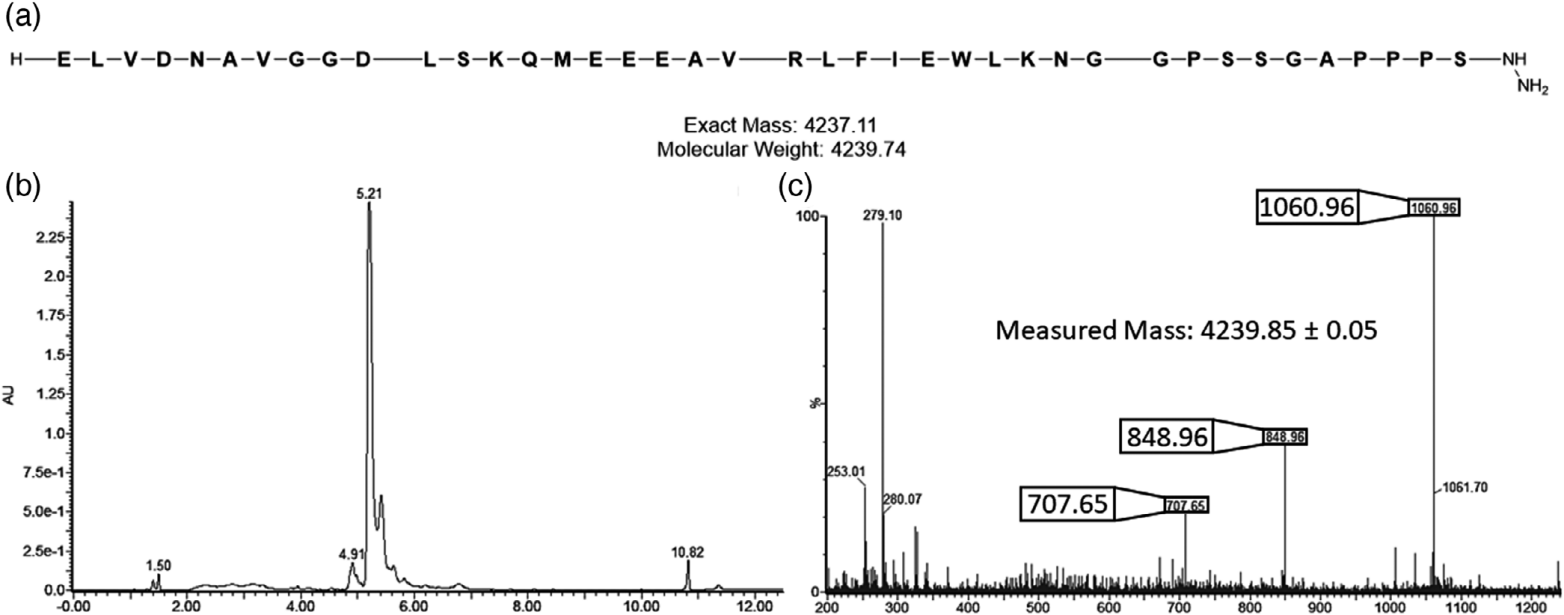
Synthesis of a 40‐mer peptide on Tentagel® XV Fmoc‐NHNH‐Trityl resin. (a) Sequence and calculated mass of the GLP‐1R agonist peptide P5. (b) UV trace of crude P5 peptide. (c) Combined mass spectrum of the crude material (simulated direct infusion)

To demonstrate the capacity of Fmoc‐NHNH‐Trityl resins for the synthesis of large peptides, the 40 amino acid GLP1‐R agonist peptide P5^[^ ^51^ ^]^ was synthesized at 0.1 mmol scale using HATU/DIEA conditions on a Fmoc‐NHNH‐Trityl Tentagel® XV (0.221 ± 0.007 mmol/g). The resulting 924 mg of resin were cleaved with 20 ml of peptide cleavage cocktail M, after precipitation from ether the peptide analyzed by LC–MS (Figure 5) and purified by automated prep‐LCMS. The peptide was recovered in 17% yield from resin loading (calculated by mass based on the penta‐trifluoroacetate salt) and analyzed by LC–MS TOF (Figures S4–S8).

## 4 CONCLUSIONS

9‐Fluorenylmethyl carbazate has been developed as a reagent for high stability loading of PEGylated trityl chloride resins pursuant to synthesis of high value peptide C‐terminal hydrazides. Fmoc‐NHNH‐Trt Tentagel® XV resins fully retained their loadings for at least 1 month under a variety of dry and solvated storage conditions, both frozen and at room temperature, and without the need for inert atmosphere. This robust linker has enabled the development of a universal and reliable procedure for regeneration, loading, drying, and storage of Fmoc‐NHNH‐Trt resins, including PEGylated resins, to produce hydrazide peptides. The competence of these resins for synthesis of long peptides was demonstrated by the production of the 40‐amino acid “P5” peptide in a 17% yield by room temperature SPPS without specific efforts to optimize synthetic conditions. We hope this will encourage more groups to explore the myriad applications of hydrazide containing peptides, and that resin manufacturers might make available trityl chloride resins that are preloaded with Fmoc‐NHNH_2_ to make these methods available to the broadest possible scope of researchers.

## CONFLICT OF INTEREST

Philip E. Dawson serves as an Advisory Board member of *Peptide Science*, and was excluded from both the peer‐review process and all editorial decisions related to the publication of this article.

## Supporting information


**Appendix S1**Supporting Information

## Data Availability

The data that supports the findings of this study are available in the supplementary material of this article.
