# Supplementary material for: A shelf stable Fmoc hydrazine resin for the synthesis of peptide hydrazides
Source: Pept Sci (Hoboken). 2022 Apr 16;114(5):e24268. doi: 10.1002/pep2.24268 (PMC9662761; doi:10.1002/pep2.24268)
Supplement: Supplementary file 1 — Appendix S1Supporting Information [file PEP2-114-e24268-s001.pdf]

Supporting Information for:

A Shelf Stable Fmoc Hydrazine Resin for the Synthesis of Peptide Hydrazides

Michael J. Bird and Philip E. Dawson

Department of Chemistry, Scripps Research, La Jolla, CA 92037, United States

Contact address: Dawson@scripps.edu

Table of Contents

On the drying of Peptide Resins 2

Supplementary Figures 2

Figure S1 2

Figure S2 3

Figure S3 3

Supplementary Tables 4

Table S1 4

Table S2 4

Table S3 5

Table S4 5

Table S5 6

Table S6 6

Table S7 7

Table S8 7

Analytical Data for Synthesis of P5 Hydrazide 8

Figure S4 8

Figure S5 8

Figure S6 8

Figure S7 9

Figure S8 9

On the drying of Peptide Resins

We would like to further emphasize the necessity of appropriately drying peptide resins following loading in order to achieve both an accurate measure of the loading and to allow for long term stable storage of the resins. In particular, our experience with PEGylated resins like Tentagel® and ChemMatrix® has shown that drying with dichloromethane (DCM) alone is insufficient. Tentagel® dried from DCM alone will typically be very sticky and hard to weigh out, and we have found that loading tests of DCM dried resins will return lower values than the same batch of resin dried with diethyl ether. Moreover, PEG-bearing Fmoc-NHNH-Trt resins dried with DCM alone without the addition of a final wash with diethyl ether appear to be less stable. Prior to storage of an Fmoc-NHNH-Trt Tentagel® or ChemMatrix® resin the resin should be flow washed thoroughly with diethyl ether over a vacuum manifold to ensure full clearance of dimethylformamide (DMF) and DCM from the resin. This also ensures the resin is fully “shrunk” from the solvated “swollen” state. This change should be visually apparent to the researcher. It is important to note that researchers should take care to regularly empty or dispose of waste containers into which diethyl ether is flowed, to mitigate the risk of dangerous peroxide buildups.

Supplementary Figures

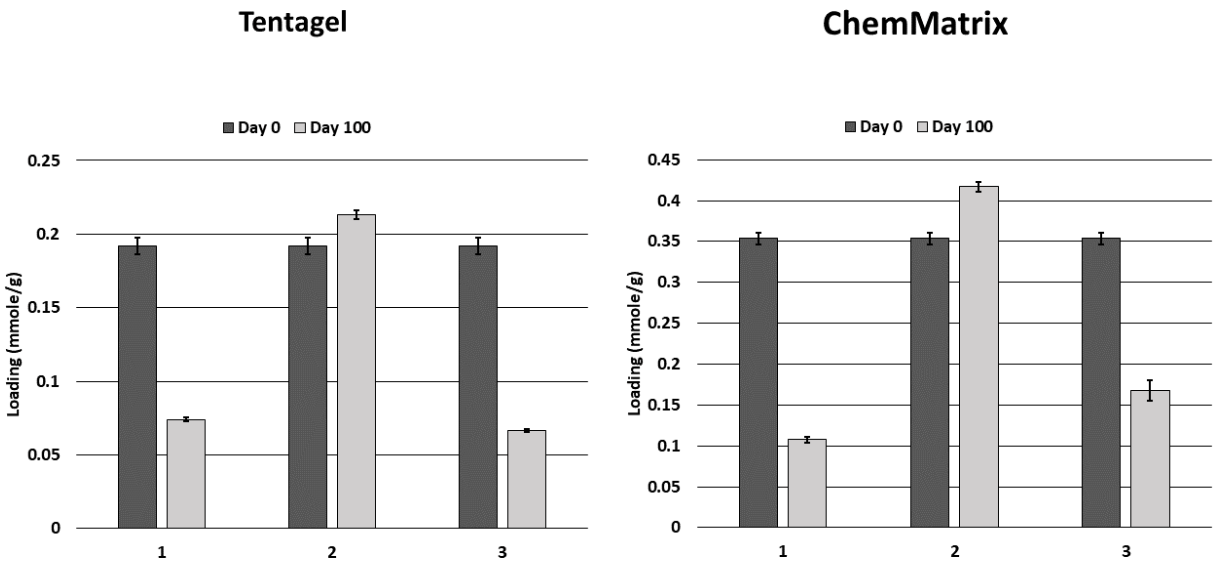

Figure S1: Stability of Tentagel XV and ChemMatrix resins stored for 100 days in a -10°C freezer as (1) Cl-Trityl under nitrogen, (2) Fmoc-NHNH-Trityl under nitrogen, (3) NH_2NH-Trityl in acetone. All error bars show ± 1 standard deviation for the triplicate loading tests. Dark grey is day 0, light grey is day 100. Instrument failure during the experimental period meant the day 0 and day 100 measurements were made on different instruments.

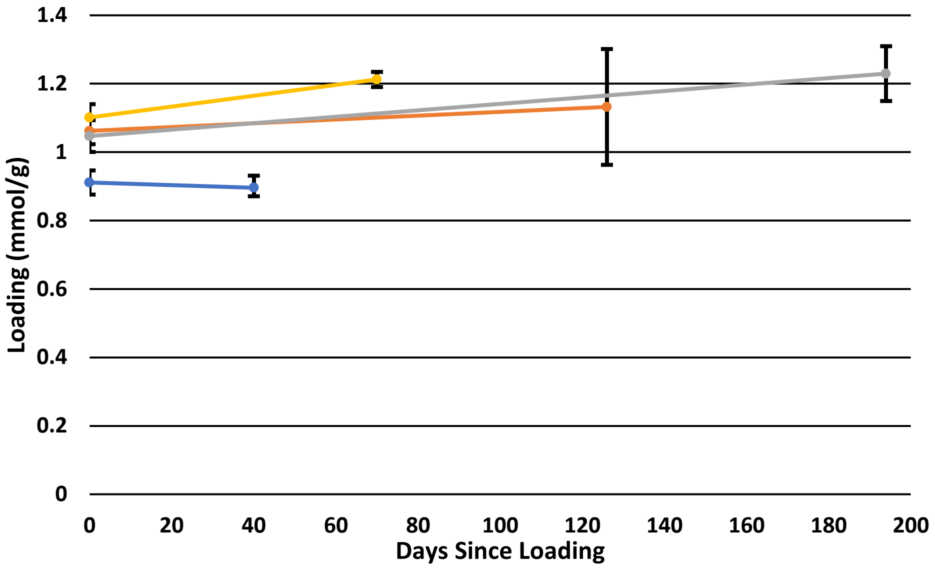

Figure S2: Retention of loading over time (measured as per Methods section 2.3) of four independent samples of Fmoc-NHNH-2-Cl-Trityl polystyrene resin (200-400 mesh, Bachem). All error bars show ± 1 standard deviation for the triplicate loading tests.

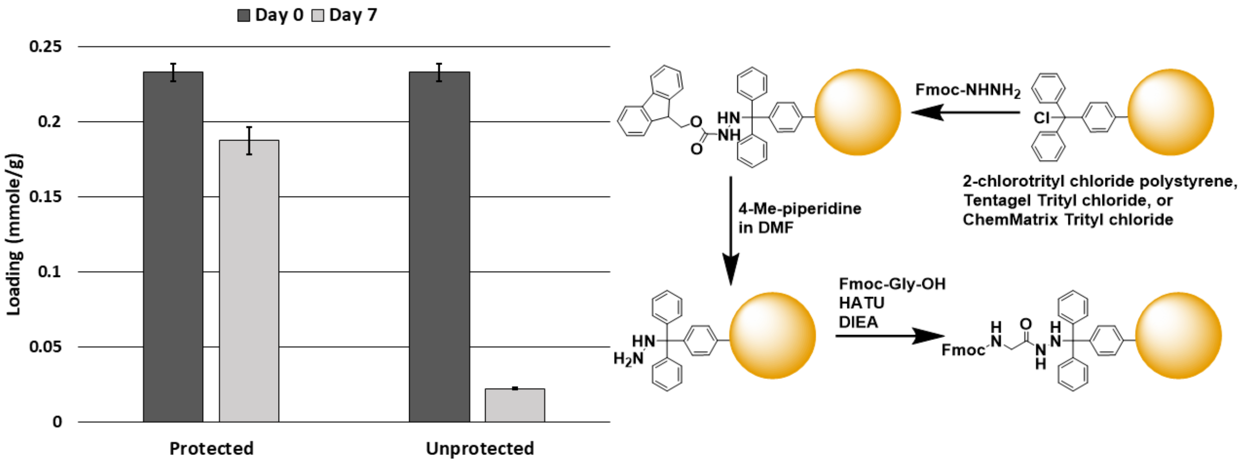

Figure S3: Stability of Tentagel XV resin stored in a vacuum desiccator for 7 days as either Fmoc-NHNH-Trityl (protected) or NH_2NH-Trityl (unprotected). All error bars show ± 1 standard deviation for the triplicate loading tests. Dark grey is day 0, light grey is day 7.

Supplementary Tables

Table S1: Absorbance data for Tentagel in Figure S1

Time (Days)	Condition	Resin Mass (mg)	A_{301}	Loading (mmole/g)	Average	Standard Deviation
**0**	N/A	12.7	0.187	0.191	0.192	0.006
		13	0.188	0.187		
		25.2	0.386	0.198		
**100**	1	25.7	0.147	0.074	0.074	0.001
		18.9	0.11	0.075		
		21.6	0.121	0.073		
**100**	2	22.2	0.37	0.216	0.213	0.003
		25.7	0.423	0.213		
		23.2	0.376	0.210		
**100**	3	21.9	0.113	0.067	0.067	0.001
		26.5	0.134	0.065		
		21.5	0.112	0.067		

Table S2: Absorbance data for ChemMatrix in Figure S1

Time (Days)	Condition	Resin Mass (mg)	A_{301}	Loading (mmole/g)	Average	Standard Deviation
**0**	N/A	15.1	0.42	0.360	0.353	0.007
		16.5	0.451	0.354		
		15.7	0.42	0.346		
**100**	1	16.5	0.134	0.105	0.107	0.004
		17.3	0.141	0.106		
		17.9	0.154	0.111		
**100**	2	18.6	0.592	0.412	0.417	0.006
		17.9	0.574	0.415		
		18	0.589	0.424		
**100**	3	15.5	0.217	0.181	0.168	0.013
		17.2	0.22	0.166		
		14.6	0.176	0.156		

Table S3: Absorbance data for Figure 2.

Time (Days)	Condition	Resin Mass (mg)	A_{301}	Loading (mmole/g)	Average	Standard Deviation
**0**	N/A	26.6	0.452	0.220	0.228	0.008
		19.7	0.358	0.235		
		17.4	0.309	0.230		
**28**	A	28.3	0.066	0.030	0.030	0.001
		24.7	0.059	0.031		
		30.4	0.07	0.030		
**28**	B	26	0.458	0.228	0.222	0.011
		40	0.707	0.229		
		25	0.406	0.210		
**28**	C	24.9	0.433	0.225	0.227	0.002
		29.8	0.529	0.230		
		38.1	0.666	0.226		
**28**	D	20.3	0.355	0.226	0.233	0.006
		30.5	0.553	0.235		
		31.5	0.579	0.238		
**28**	E	30	0.541	0.234	0.232	0.001
		33.1	0.592	0.232		
		29.2	0.523	0.232		

Table S4: Absorbance data for Figure S3

Time (Days)	Condition	Resin Mass (mg)	A_{301}	Loading (mmole/g)	Average	Standard Deviation
**0**	N/A	20.3	0.355	0.226	0.233	0.006
		30.5	0.553	0.235		
		31.5	0.579	0.238		
**7**	Protected	44.4	0.608	0.177	0.188	0.009
		32.9	0.493	0.194		
		51.5	0.761	0.191		
**7**	Unprotected	45.6	0.078	0.022	0.022	0.001
		42.1	0.071	0.022		
		50.4	0.09	0.023		

Table S5: Absorbance data for Figure 3

Time (Days)	Condition	Resin Mass (mg)	A_{301}	Loading (mmole/g)	Average	Standard Deviation
**0**	N/A	22.9	0.387	0.219	0.221	0.007
		16.8	0.28	0.216		
		15.8	0.28	0.229		
**30**	A	33.9	0.591	0.226	0.229	0.008
		44.8	0.769	0.222		
		45.3	0.832	0.238		
**30**	B	31.3	0.546	0.226	0.224	0.002
		52	0.9	0.224		
		38.5	0.659	0.222		
**30**	C	37.4	0.64	0.222	0.219	0.006
		43.6	0.753	0.224		
		39.1	0.642	0.213		
**30**	D	41.3	0.714	0.224	0.225	0.012
		39.8	0.729	0.237		
		50.3	0.832	0.214		
**30**	E	34.3	0.591	0.223	0.224	0.002
		37.6	0.657	0.226		
		32.9	0.568	0.224		
**30**	F	55.2	0.957	0.224	0.228	0.003
		42.2	0.742	0.228		
		38.1	0.678	0.230		
**30**	G	39.5	0.671	0.220	0.228	0.015
		38	0.721	0.246		
		38	0.642	0.219		
**30**	H	36.9	0.634	0.222	0.227	0.011
		45.9	0.779	0.220		
		37.9	0.702	0.240		

Table S6: Absorbance Data for Figure 4 Sample A

Time (Days)	Resin Mass (mg)	A_{301}	Loading (mmole/g)	Average	Standard Deviation
**0**	23.4	0.381	0.211	0.209	0.003
	22.7	0.37	0.211		
	19.8	0.314	0.205		
**30**	11.6	0.186	0.208	0.206	0.008
	7.7	0.127	0.214		
	8.5	0.13	0.198		

Table S7: Absorbance Data for Figure 4 Sample B

Time (Days)	Resin Mass (mg)	A_{301}	Loading (mmole/g)	Average	Standard Deviation
**0**	19.1	0.316	0.214	0.213	0.002
	29.7	0.489	0.213		
	24.7	0.402	0.211		
**30**	15	0.236	0.204	0.204	0.001
	16.3	0.258	0.205		
	11.7	0.184	0.204		

Table S8: Absorbance Data for Figure S3

Experiment	Time (Days)	Resin Mass (mg)	A_{301}	Loading (mmole/g)	Average	Standard Deviation
**Blue**	0	8.25	0.601	0.943	0.911	0.035
		11.86	0.8	0.873		
		5.59	0.396	0.917		
	40	9.3	0.663	0.923	0.896	0.025
		8.49	0.584	0.891		
		7.91	0.534	0.874		
**Orange**	0	6.22	0.52	1.083	1.062	0.039
		10.64	0.893	1.087		
		13.67	1.074	1.017		
	126	8	0.816	1.321	1.132	0.169
		8.6	0.719	1.083		
		7.3	0.56	0.993		
**Grey**	0	9.5	0.742	1.011	1.047	0.046
		5.9	0.501	1.100		
		9	0.716	1.030		
	194	7.6	0.774	1.319	1.230	0.080
		6.8	0.611	1.163		
		8.8	0.82	1.207		
**Yellow**	0	13.6	1.128	1.074	1.101	0.039
		7.1	0.619	1.129		
		N/A	N/A	N/A		
	70	9.1	0.839	1.194	1.212	0.022
		4.7	0.438	1.207		
		7.6	0.726	1.237		

Analytical Data for Synthesis of P5 Hydrazide

H—E—L—V—D—N—A—V—G—G—D—L—S—K—Q—M—E—E—E—A—V—R—L—F—I—E—W—L—K—N—G—G—P—S—S—G—A—P—P—P—S—NH—NH_2

Exact Mass: 4237.11
Molecular Weight: 4239.74

Figure S4: Sequence and expected mass of P5 hydrazide peptide.

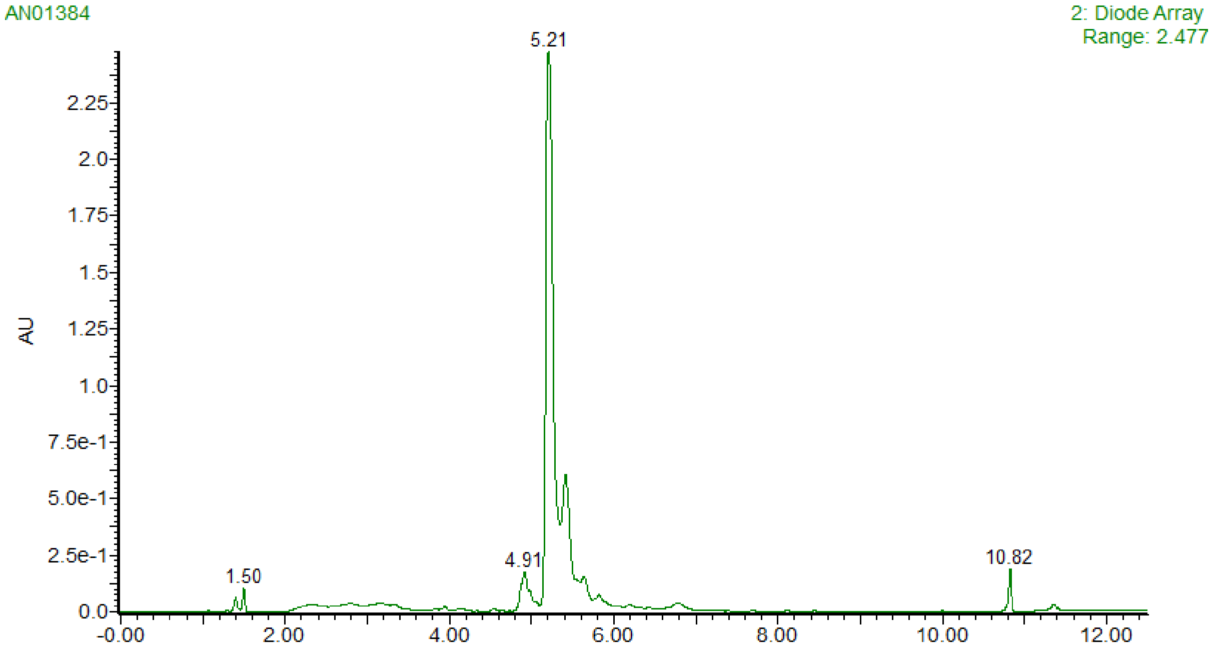

Figure S5: Crude HPLC UV trace of P5 hydrazide peptide.

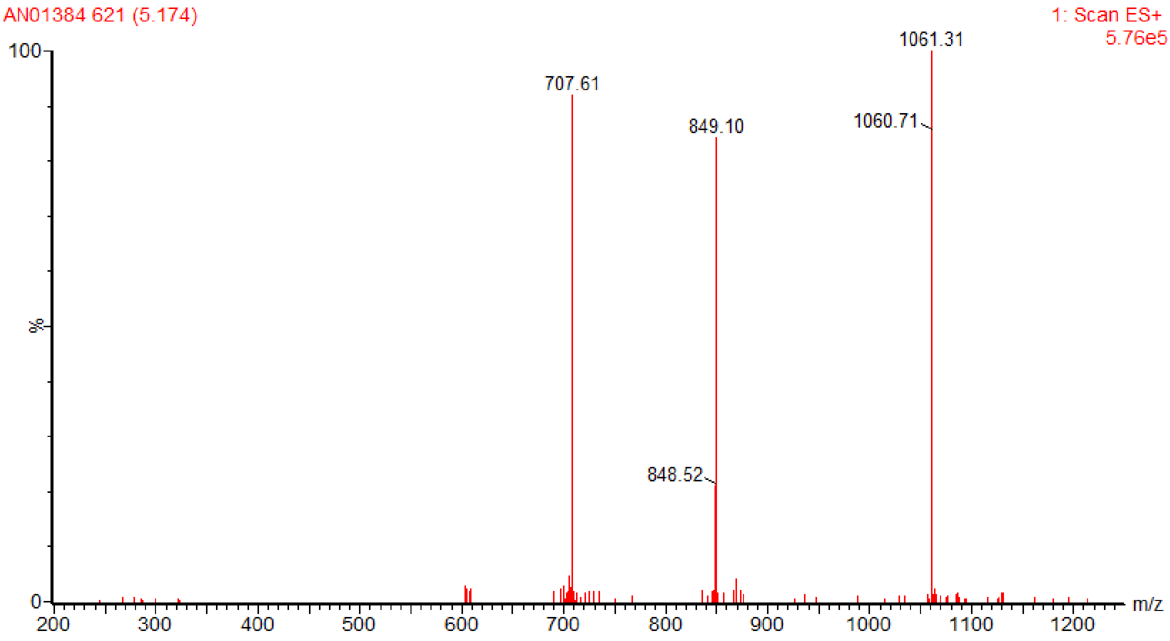

Figure S6: Crude TOF-MS of P5 hydrazide peptide under the primary peak.

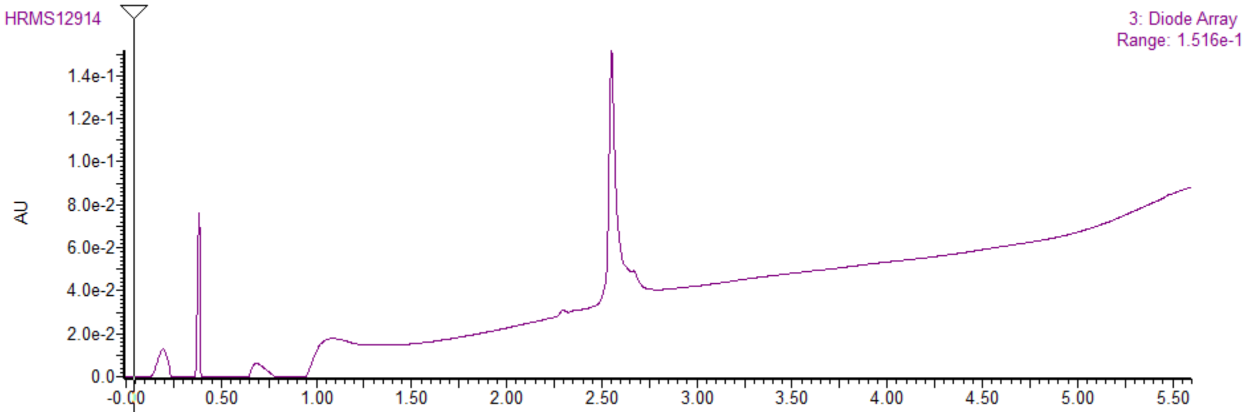

Figure S7: HPLC UV Trace of purified P5 hydrazide peptide.

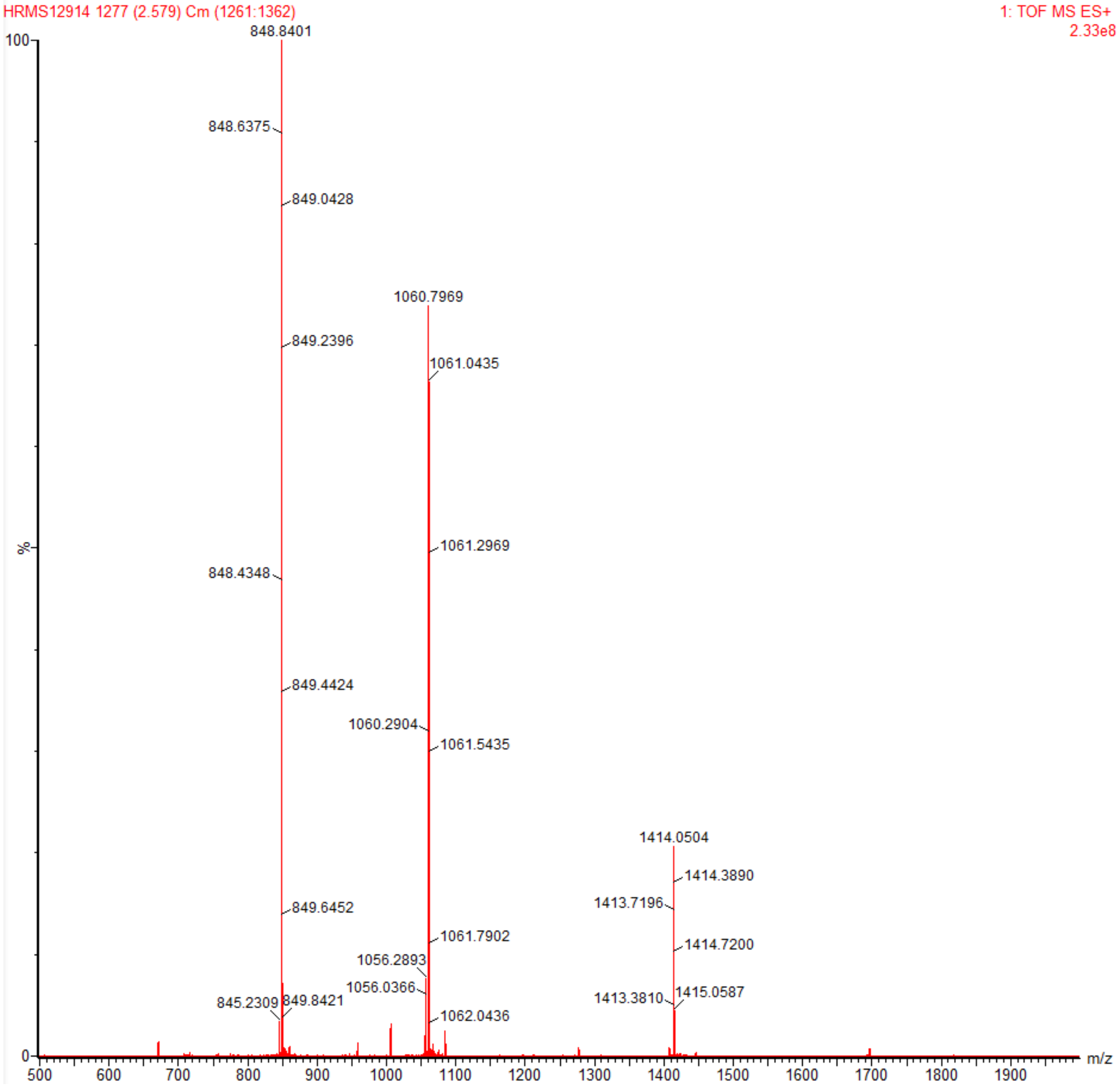

Figure S8: Combined TOF-MS of purified P5 hydrazide peptide.
